# Rapid and specific detection of *Streptococcus suis* serotype 2 using a RPA–PfAgo system coupled with fluorescence and lateral flow dipstick

**DOI:** 10.3389/fvets.2025.1748435

**Published:** 2026-01-13

**Authors:** Kexin Wang, Xujie Zhao, Jingjing Li, Mingzhu Zhou, Bingze Jiao, Yilin Bai, Zhanwei Teng, Meinan Chang, Yueyu Bai, Jianhe Hu, Ke Ding, Xiaojing Xia

**Affiliations:** 1 College of Animal Science and Veterinary Medicine, Henan Institute of Science and Technology, Xinxiang, China; 2 Ministry of Education Key Laboratory for Animal Pathogens and Biosafety, Zhengzhou, China; 3 Laboratory of Indigenous Cattle Germplasm Innovation, School of Agricultural Sciences, Zhengzhou University, Zhengzhou, China; 4 Animal Health Supervision of Henan Province, Bureau of Animal Husbandry of Henan Province, Zhengzhou, China

**Keywords:** fluorescence detection, lateral flow dipstick, PfAgo, point-of-care testing, rapid diagnosis, recombinase polymerase amplification, *Streptococcus suis* serotype 2

## Abstract

**Objective:**

To develop and validate dual detection platforms integrating recombinase polymerase amplification (RPA) with Pyrococcus furiosus Argonaute (PfAgo) for the rapid and specific identification of *Streptococcus suis* serotype 2.

**Methods:**

The conserved *cps2J* gene was selected as the molecular target. Key RPA parameters and PfAgo reaction conditions were systematically optimized, including temperature, reaction time, MnCl_2_ concentration, gDNA design and probe concentration. Specificity and sensitivity were evaluated using plasmid dilutions and multiple *S. suis* serotypes together with other common swine pathogens. A total of 41 clinical samples were also tested and compared with the national standard PCR assay (GB/T 19915.3–2005).

**Results:**

Two assay formats were established: real-time fluorescence system (RPA-PfAgo-RTF) and lateral flow dipstick system (RPA-PfAgo-LFD). The RPA-PfAgo-RTF assay achieved a detection limit of 10^0^ copies/μL, while the RPA-PfAgo-LFD assay detected 10^2^ copies/μL. Both formats showed high specificity without cross-reactivity. Among 41 field samples, six were SS2-positive, and results showed 100% agreement with the reference PCR method. Total detection time for either assay was < 1 h.

**Conclusion:**

Both assay formats provide rapid, sensitive, and accurate tools for SS2 detection suitable for laboratory use and on-farm point-of-care testing.

## 1 Introduction

*Streptococcus suis* (SS) is a significant zoonotic pathogen that causes major economic losses in the pig industry and poses a potential threat to human health (1, 2). Among the 29 identified serotypes, serotype 2 (SS2) is the most prevalent and virulent. It causes severe diseases in pigs, including sepsis, meningitis, arthritis, and pneumonia, and can lead to serious infections in humans (3, 4). Therefore, rapid and accurate detection of SS2 is essential for effective disease control, reduction of economic losses, and prevention of zoonotic transmission (5, 6).

Traditional diagnostic methods, such as bacterial culture and biochemical testing, often lack adequate sensitivity and specificity and are time-consuming (7). Recent advances in molecular biology diagnostics—such as real-time PCR, loop-mediated isothermal amplification (LAMP), and CRISPR/Cas systems—have enabled more rapid and accurate detection of *S. suis* (8–10). However, these techniques require specialized equipment and trained personnel (11, 12), which limit their use in field or resource-poor settings. Furthermore, antimicrobial resistance in SS2 is increasing and some isolates show strong resistance to commonly used antibiotics, complicating disease control (13, 14). Therefore, the development of a rapid detection method for SS2 with high sensitivity, strong specificity, and cost-effectiveness is particularly important. This would not only improve the efficiency of SS2 diagnosis but also provide a reference for the rational use of antibiotics, thereby reducing the emergence of resistant strains.

Recently, the RPA-PfAgo system has emerged as a promising tool for rapid bacterial detection (15). This method first use RPA to amplify the target nucleic acid at 37–42 °C, after which PfAgo, guided by short DNA oligonucleotides, cleaves the products at 85–95 °C to generate secondary signals (16–18). Unlike CRISPR/Cas systems, PfAgo does not require protospacer adjacent motif (PAM) sequences and can achieve single-base resolution through guide design, making it useful for distinguishing resistance loci and closely related species (19, 20). Several readout formats, such as agarose gel electrophoresis, real-time fluorescence (18), lateral flow dipstick (LFD) (21, 22), and digital platforms (23), have been developed, achieving detection limits ranging from 10^0^ to 10^2^ copies per reaction. The method has been successfully applied to a variety of sample types, such as milk, serum, urine, and clinical swabs (18, 24).

In this study, we developed two RPA-PfAgo assays targeting the *cps2J* gene of SS2: a real-time fluorescence assay and a LFD assay. Key reaction parameters were optimized, and their sensitivity and specificity were thoroughly evaluated. The performance of the assays was further validated using clinical samples and compared with that of the Chinese national standard PCR method. Subsequently, the detection systems were applied to the analysis of clinical tissue samples to confirm their feasibility and practicality for on-site diagnosis. This study presents an efficient, user-friendly, and field-deployable technical approach for the early and rapid detection of SS2, which holds significant potential for the effective prevention and control of SS2 infections.

## 2 Methods

### 2.1 Bacterial strains

The bacterial strains used in this study included SS1 (JZLQ036), SS2 (CVCC606, CVCC1941, JZLQ022, ZY05719, 05ZYH33, and JZLQ019), SS7 (JZLQ034), SS9 (JZLQ035), *Actinobacillus pleuropneumoniae* (APP, CVCC259), *Pasteurella multocida* (C44-1), *Glaesserella parasuis* (GPS, Isolated strain), *Salmonella* (CVCC541), Enteropathogenic *Escherichia coli* (EPEC, Isolated strain), *Staphylococcus aureus* (*S. aureus*, ATCC49525) and *Aeromonas hydrophila* (*A. hydrophila*, AH-1). All strains were obtained and preserved in the Laboratory of Preventive Veterinary Medicine, Henan Institute of Science and Technology.

### 2.2 Reagents

The bacterial genomic DNA kit and pfAgo were purchased from Absin (Shanghai) Biotechnology Co., Ltd. The TwistAmp Basic kit was obtained from TwistDx (United Kingdom). The PCR Master Mix, plasmid DNA extraction kit, and 4S GelRed nucleic acid staining solution were supplied by Sangon Biotech (Shanghai) Co., Ltd. The flow measurement chromatography test strips were acquired from Milenia Biotec GmbH (Germany), and the gel recovery kit was sourced from Servicebio Biotechnology (Wuhan) Co., Ltd. The 20% TBE-PAGE gel preparation solution was purchased from Coolaber Technology (Beijing) Co., Ltd. BHI, TSB, and LB media were obtained from Solarbio Technology (Beijing) Co., Ltd. TAE (50X) was procured from Beyotime Biomedical Technology (Shanghai) Co., Ltd, the 1,000 bp DNA marker was acquired from BaKaRa Medical Biology Technology (Beijing) Co., Ltd. LAR AGAROSE/agarose was obtained from baygene Biotechnology (Shanghai) Co., Ltd.

### 2.3 Preparation of bacterial DNA

The frozen bacterial strains were thawed and streaked onto solid culture medium. Individual colonies were subsequently selected and cultured in liquid medium. Specifically, THB medium was used for the cultivation of SS, BHI medium for APP, TSB medium for Pm and GPS, and LB medium for *A. hydrophila*, EPEC, *S. aureus*, and *Salmonella*. Genomic DNA was extracted from bacteria in the logarithmic growth phase using a bacterial genomic DNA extraction kit. The mass concentration and purity of the extracted DNA from each strain were measured using an ultra-micro nucleic acid and protein concentration analyzer, and the samples were stored at −80°C for future use.

### 2.4 Design and screening of RPA primers, gDNA, and probes, and construction of the *cps2J* plasmid

The conserved SS2 *cps2J* gene (Accession Number: DQ410853.1) was selected as the detection target, and primers and probes were designed following the TwistAmp™ Basic kit (TwistDX, UK) guidelines using Primer 5.0. The RPA primers, gDNAs, fluorescent ssDNA reporter probe (5′6-FAM-ssDNA-BHQ1-3′), and lateral flow assay reporter probe MB (5′6-FAM-ssDNA-Biotin-3′) were synthesized by Sangon Biotech (Shanghai, China). All sequence information is listed in Table 1. The recombinant plasmid was constructed using the pGEM-T Easy vector (Promega, Beijing), and the positive plasmid was designated as pGEM-T-*cps2J*, which served as the DNA template standard.

**Table 1:** Primer and probe sequence.

**Primer name**	**Sequence(55^′^-33^′^)**
CPS2J-212F	GGAATACGCAGAGCAAGATGGTAGAATAAA
CPS2J-212R	AAAAGTAGCAAGTAACCCTCCCGACAAATC
gDNA1-1	GTCCTTATACACCTGT
gDNA1-2	ATTGTTGACGGCAACA
gDNA1-3	TTGTTGAGTCCTTATA
gDNA1-4	TTTAAACAGGTGTATA
gDNA1-5	TAAACAGGTGTATAAG
gDNA1-6	GGTGTATAAGGACTCA
gDNA1-7	AATGTTGCCGTCAACA
gDNA2	ATTGTTGACGGCAACA
gDNA3	GACTCAACAATGTTGC
RTF-MB	6-FAM-CGCACCGCAACATTGTTGAGTCGGTGCG-BHQ1
LFD-MB	FAM-GCAACATTGTTGAGTC-Biotin
SSDNA	TAGATTCTGATGATATTGTTGACGGCAACATTGTTGAGTCCTTATACACCTGTTTAAAAG

### 2.5 Establishment and optimization of RPA detection method

According to the instructions of the TwistAmp^®^ RPA kit (TwistDx, Cambridge, UK), a 50 μL reaction system was established for RPA amplification. The reaction mixture consisted of 29.5 μL rehydration buffer, 2.5 μL of 280 mM magnesium acetate, 2.4 μL of 10 μM forward and reverse primers, 2 μL of DNA, and 11.2 μL of nuclease-free water. The mixture was gently mixed, briefly centrifuged, and then incubated at 39 °C for 30 min. Amplification products were analyzed by 2% agarose gel electrophoresis.

To maximize the sensitivity of the Basic-RPA reaction system, a single-factor experimental design was employed to optimize key parameters, including reaction temperature and amplification time. The temperature gradient was set at six incremental levels ranging from 25 to 45 °C (at 5 °C intervals), covering the manufacturer's recommended operating range of 39 ± 5 °C. Based on the optimal temperature identified, a time gradient ranging from 5 to 40 min (at 5 min intervals) was further tested. The brightness of the amplified bands was analyzed to determine the optimal reaction conditions.

### 2.6 Verification of PfAgo cutting principles

To evaluate the cleavage activity of the PfAgo protein, the following reaction system was established: 10 μL of 10 μM ssDNA, 1 unit of PfAgo enzyme, 2 μL of 100 μM 5′-phosphorylated gDNA, 2.5 μL of 10 × reaction buffer, and 1 μL of 40 mM MnCl_2_. The final volume was adjusted to 25 μL with nuclease-free water. The reaction was performed in a real-time PCR instrument at a constant temperature of 95 °C for 40 min. The reaction products were analyzed by 20% TBE-PAGE to verify the cleavage activity of the PfAgo protein.

### 2.7 Optimization of RPA-PfAgo-RTF

The RPA-PfAgo-RTF reaction was performed under the optimized conditions described in section 1.5. Specifically, 2 μL of the RPA amplification product was added to the PfAgo reaction mixture, along with 2 μL each of gDNA1 (10 μM), gDNA2 (10 μM), and gDNA3 (10 μM), 1 μL of molecular beacon (MB, 10 μM), 1 μL of PfAgo endonuclease, 1 μL of MnCl_2_ (40 mM), and 2.5 μL of 10 × reaction buffer„ and the final volume was adjusted to 25 μL with nuclease-free water. The reaction was carried out in a real-time PCR instrument at a constant temperature of 95 °C for 40 min. To achieve optimal reaction performance, single-factor optimization was conducted for MnCl_2_ concentration (0.5, 1, 1.6, 2, 2.4, and 3.2 mM), probe concentration (0.2, 0.4, 0.6, 0.8, 1, and 1.2 μM), gDNA concentration (0.2, 0.5, 0.8, 1.2, and 1.5 μM), reaction temperature (85, 89, 92, 95, 97, and 99 °C), and reaction time (10, 20, 30, 40, 50, and 60 min).

### 2.8 Establishment of the RPA-PfAgo-LFD detection system

Based on the establishment of the RPA-PfAgo detection system, a visual detection method was developed by integrating Lateral Flow Dipstick (LFD) technology. In the RPA-PfAgo-RTF detection system developed in section 1.7, the original RTF-MB probe was replaced with the LFD-MB probe to construct the RPA-PfAgo-LFD detection system. Following the RPA-PfAgo reaction, 100 μL of nuclease-free water was added to the reaction mixture, and the test strip was then inserted. The system was incubated at room temperature for 5 min before the strip was removed and photographed. During LFD detection, the biotin-labeled probe binds to streptavidin on the test line after cleavage by PfAgo, while gold-labeled antibodies accumulate at the control line to confirm strip validity. The criteria for interpreting the test results are as follows: In a positive sample, the probe is cleaved, allowing both the detection line (T line) and the control line (C line) to appear on the test strip. In contrast, in a negative sample, the probe remains intact, resulting in the absence of a band on the test line while the control line remains visible. If neither the test line nor the control line appears, the result is considered invalid.

### 2.9 Evaluation of specificity and sensitivity

The *cps2J* positive plasmid was serially diluted tenfold (from 1 × 10^6^ to 1 × 10^0^ copies/μL) and subjected to isothermal amplification using the optimized RPA method. The amplification products were then applied to the optimized RPA-PfAgo-RTF and RPA-PfAgo-LFD detection systems to evaluate the sensitivity of the fluorescence-based assay. Genomic DNA from *Streptococcus suis*, APP, Pm, GPA, *Salmonella*, EPEC, *S. aureus*, and *A. hydrophila* was used as templates, and detection was performed according to the methods described in sections 1.7 and 1.8 to assess the specificity of these two detection methods.

### 2.10 Clinical samples testing

To evaluate the reliability of the methods established in this study, a total of 41 samples were collected from suspected *Streptococcus suis* serotype 2–infected pigs from pig farms of different scales in Henan Province. The samples, including tonsils, lungs, and blood, were subjected to DNA extraction and subsequent analysis. Simultaneously, all samples were tested by PCR according to the national standard (GB/T 19915.3-2005).

## 3 Results

### 3.1 Principle of the RPA-PfAgo detection system

**Figure 1:**
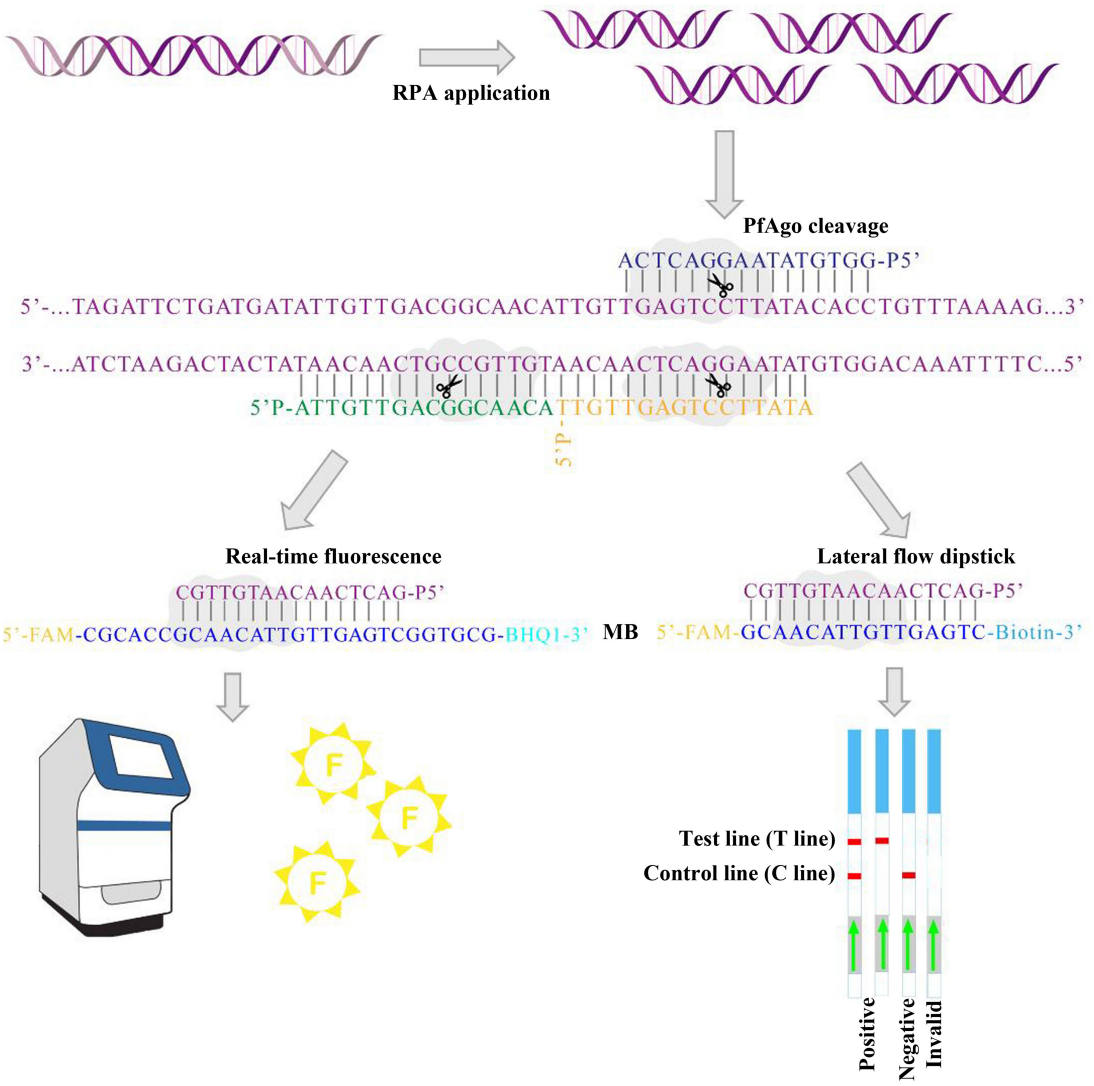
Principle of the RPA- PfAgo method.

In this study, we combined RPA-Basic amplification with PfAgo-mediated cleavage to establish a rapid, efficient, and highly sensitive nucleic acid detection system for Streptococcus suis serotype 2 (Figure 1). Genomic DNA was first extracted from samples using a commercial DNA extraction kit. The conserved *cps2J* gene was then amplified by RPA under isothermal conditions to generate double-stranded target products. The RPA products were subsequently added to the PfAgo reaction mixture containing three 5′-phosphorylated guide DNAs (gDNAs), PfAgo enzyme, and a molecular beacon (MB) probe, followed by incubation at 95 °C for 40 min. During the reaction, the primary gDNAs guide PfAgo to perform an initial cleavage of the RPA product, producing 5′-phosphorylated single-stranded DNA fragments. These fragments act as “secondary gDNAs” and direct PfAgo to cleave the complementary MB probe. As the MB carries a fluorophore and a quencher at its termini, PfAgo-mediated cleavage separates the two groups and releases a fluorescence signal for real-time detection. When an LFD-MB probe is used, cleavage enables visual detection via lateral flow strips: positive samples display both the test (T) line and control (C) line, whereas negative samples show only the C line; the absence of either line is considered invalid. This dual-cleavage, dual-readout strategy markedly enhances detection sensitivity, specificity, and operational flexibility.

### 3.2 Design and screening of RPA primers

**Figure 2:**
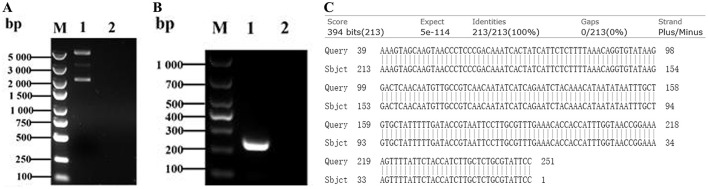
Design and screening of RPA primers. **(A)** Electrophoretic profile of the recombinant plasmid. **(B)** PCR amplification results of the target sequence analyzed by electrophoresis. **(C)** Sequence alignment and comparison outcomes.

In this study, the conserved *cps2J* gene of Streptococcus suis was selected as the target for establishing a rapid detection method. Following standard RPA primer design principles, five pairs of SS2–specific primers were designed and evaluated using the pGEM-T-*cps2J* plasmid (Figure 2A), Amplification efficiency was assessed agarose gel electrophoresis. The results showed that the primer pair CPS2J-212F/CPS2J-212R successfully produced a clear target band, while no bands were observed in the negative control (Figure 2B). Sequencing and alignment confirmed that there were no mutations or deletions in the amplified sequence (Figure 2C). Therefore, the primer pair CPS2J-212F/CPS2J-212R was selected for subsequent experiments to facilitate further studies.

### 3.3 Establishment and optimization of basic-RPA

**Figure 3:**
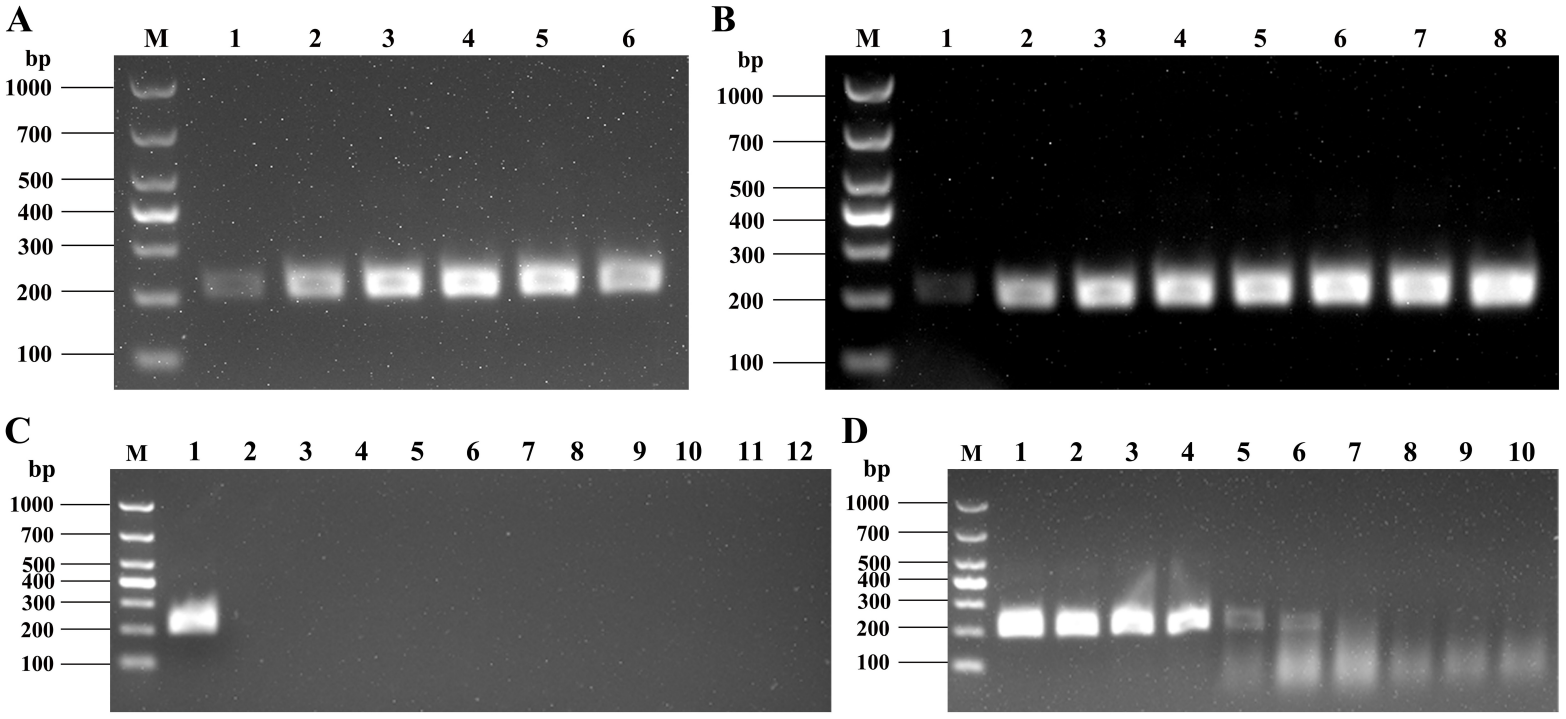
Optimization of Basic-RPA. **(A)** Optimization of RPA reaction temperature. 1–6: 25°C, 30°C, 35°C, 37°C, 39°C, 45°C. **(B)** Optimization of RPA reaction time, 1–8: 5 min, 10 min, 15 min, 20 min, 25 min, 30 min, 35 min, 40 min. **(C)** Specificity Evaluation. 1–12: Positive control, Salmonella, L. monocytogenes, GPS, Pm, A. hydrophila, and E. coli, ntc (no-template control). **(D)** Sensitivity Evaluation. 1–10: 1 × 10^0^, 1 × 10^−1^, 1 × 10^−2^, 1 × 10^−3^, 1 × 10^−4^, 1 × 10^−5^, ntc. M: DL1000 DNA Marker.

To achieve optimal amplification efficiency of the Basic-RPA assay, the reaction time and temperature were optimized. When the reaction time was fixed at 20 min and the primer concentration at 10 μM, the amplification efficiency of RPA was further evaluated under different temperatures. The results indicated that target bands were observed at 30–45 °C, with the brightest band obtained at 37 °C (Figure 3A). Therefore, 37 °C was determined to be the optimal reaction temperature for the Basic-RPA assay. The Basic-RPA reaction was further evaluated at different incubation times using a fixed temperature of 37 °C and primer concentration of 10 μM. The results showed that target bands were obtained at 10–35 min, and considering both detection speed and amplification efficiency, 20 min was selected as the optimal reaction time for the Basic-RPA assay (Figure 3B).

Using the optimized RPA reaction system and conditions, assays were performed on seven common pathogenic bacteria–APP, Pm, GPS, *Salmonella*, EPEC, *S. aureus, A. hydrophila*–as well as SS1, SS7, and SS9. The results showed that the Basic-RPA assay specifically amplified the target product only from S. suis serotype 2, while no amplification was observed for other serotypes or the tested pathogens (Figure 3C), indicating that the Basic-RPA method established in this study possesses strong specificity. To determine the sensitivity of the Basic-RPA assay, the constructed plasmid standard pGEM-T-*cps2J* was serially diluted 10-fold to different concentrations and subjected to Basic-RPA reactions. As shown in Figure 3D, the limit of detection was 1 × 10^−4^ ng/μL, which is higher in sensitivity than PCR (1 × 10^−3^ ng/μL, data not shown), demonstrating the favorable sensitivity of the assay.

### 3.4 Verification of PfAgo cleavage activity and gDNA screening

**Figure 4:**
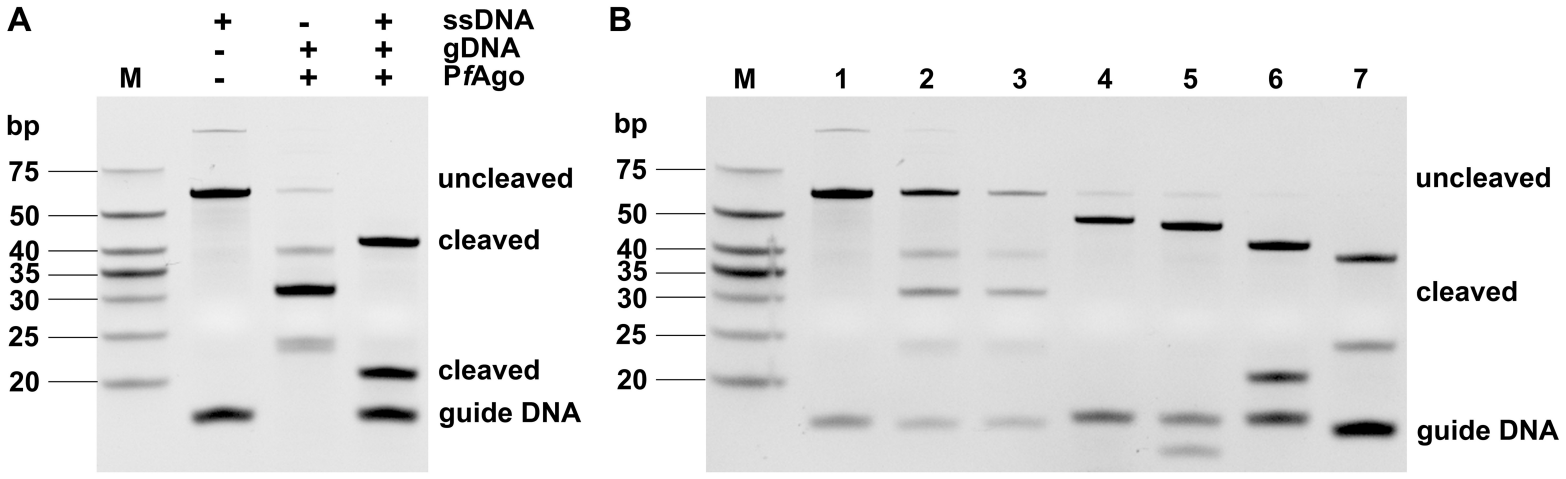
Verification of PfAgo cleavage activity **(A)** and gDNA screening **(B)**.

As shown in Figure 4A, the PfAgo protein cleaved the target DNA into two fragments of different sizes. Seven designed gDNAs were individually introduced into the PfAgo reaction system, and the reaction products were analyzed by 20% TBE-PAGE. As shown in Figure 4B, gDNA6 exhibited the most efficient cleavage activity, likely due to its favorable sequence characteristics that improve PfAgo loading and target accessibility, and was therefore selected for subsequent experiments.

### 3.5 Establishment and optimization RPA-PfAgo-RTF

**Figure 5:**
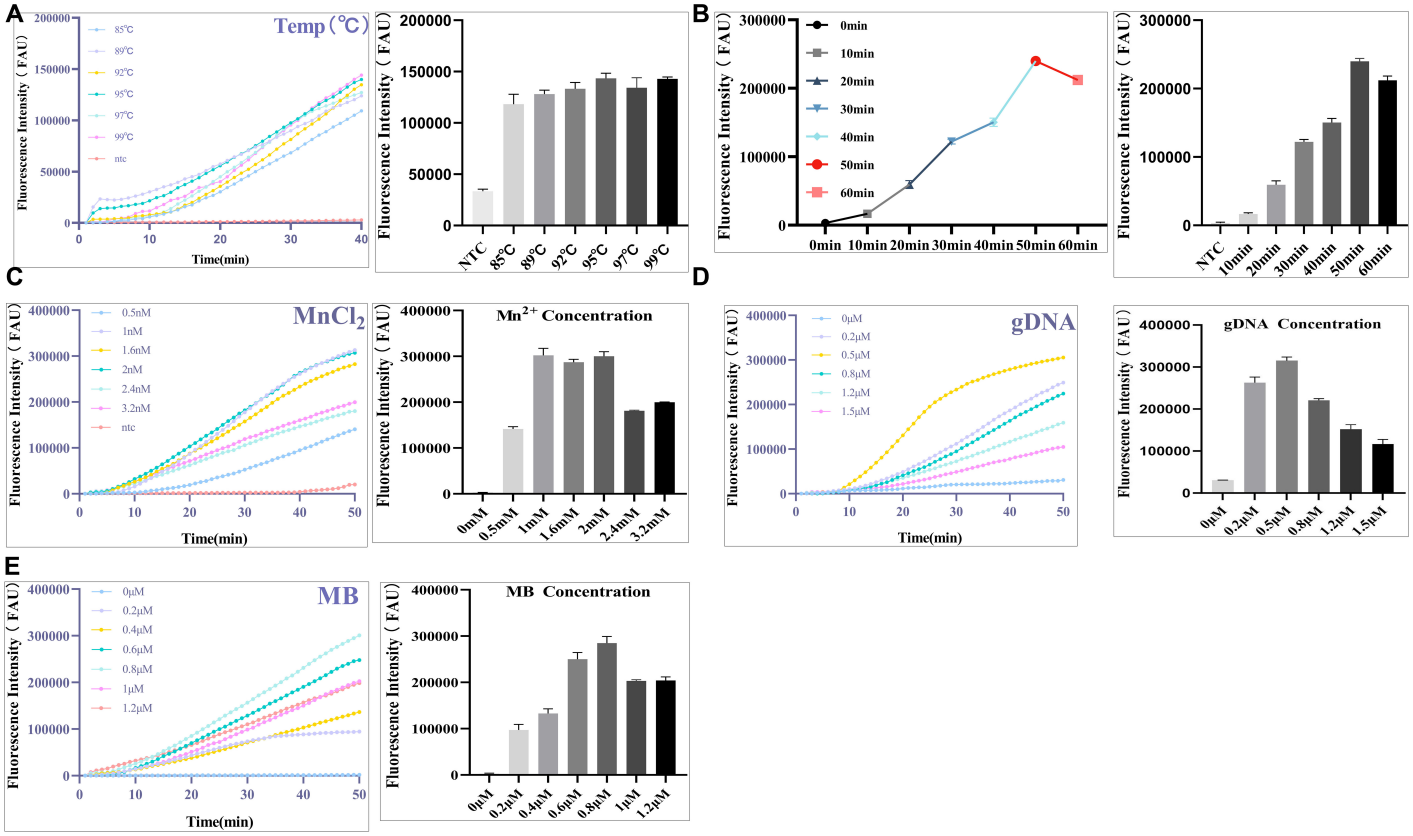
Establishment and Optimization of RPA-PfAgo-RTF reaction system**. (A)** Optimization of reaction temperature; **(B)** Optimization of reaction time; **(C)** Optimization of MnCl_2_ concentration; **(D)** Optimization of gDNA primer concentration; **(E)** Optimization of MB concentration.

The reaction system for the RPA-PfAgo fluorescence assay consisted of 2 μL gDNA, 2 units of PfAgo protein, 2.5 μL reaction buffer, 1 μL Mn^2+^, 1.5 μL molecular beacon, and 2 μL DNA template, with ddH_2_O added to a final volume of 25 μL. To improve the efficiency of the RPA-PfAgo fluorescence assay and achieve optimal detection performance, the reaction conditions (temperature and time) and system parameters (concentrations of MnCl_2_, gDNA, and molecular beacon probe) were optimized. When the reaction time was set to 40 min, with MnCl_2_ at 2 mM, gDNA at 0.8 μM, and the probe at 0.8 μM, the amplification efficiency of the RPA-PfAgo fluorescence assay was evaluated at different temperatures (89°C, 92°C, 95°C, 97°C, and 99°C). The results showed that amplification was achieved across the 85–99 °C range, with the strongest fluorescence signal observed at 95°C (Figure 5A). Therefore, 95°C was selected as the optimal reaction temperature for the RPA-PfAgo fluorescence assay. Using this approach, the optimal reaction time was determined to be 50 min (Figure 5B), the optimal MnCl_2_ concentration 1 mM (Figure 5C), the optimal gDNA concentration 0.5 μM (Figure 5D), and the optimal probe concentration 0.8 μM (Figure 5E).

### 3.6 The specificity and sensitivity of the RPA-PfAgo-RTF and the RPA-PfAgo-LFD

**Figure 6:**
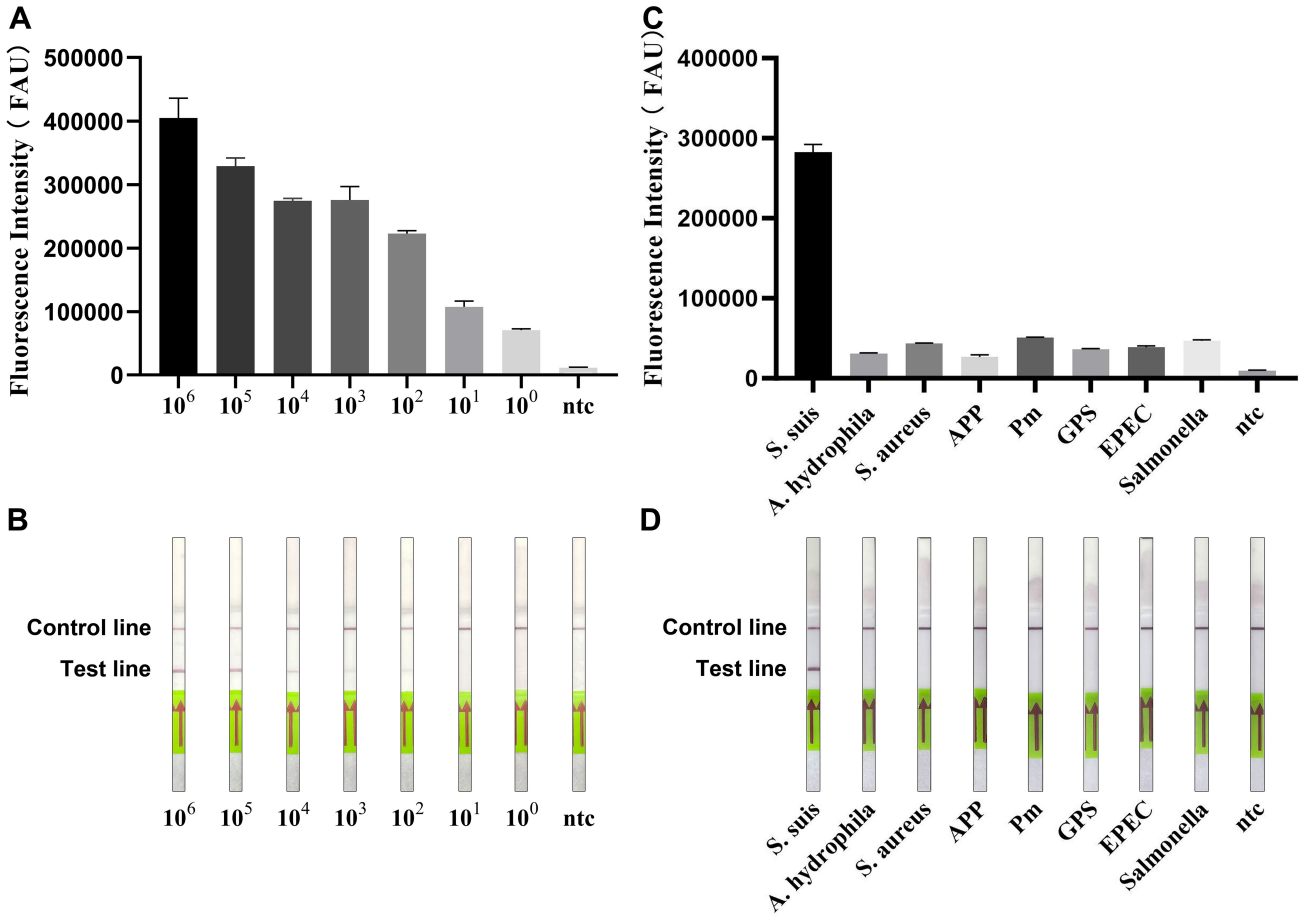
Specificity and sensitivity of RPA-PfAgo-RTF and RPA-PfAgo. **(A)** Sensitivity of RPA-PfAgo-RTF. **(B)** Sensitivity of RPA-PfAgo. **(C)** Specificity of RPA-PfAgo-RTF. **(D)** Specificity of RPA-PfAgo-LFD.

By replacing the RTF-MB probe in the RPA-PfAgo-RTF detection system developed in Section 2.4 with an LFD-MB probe, an RPA-PfAgo-LFD assay was established. The pGEM-T-*cps2J* plasmid was serially diluted 10-fold (1 × 10^6^ to 1 × 10^0^ copies/μL) and tested under optimized reaction conditions. The results showed that the limit of detection (LOD) of the RPA-PfAgo fluorescence assay was 100 copies/μL (Figure 6A), whereas that of the RPA-PfAgo-LFD strip assay was 10^2^ copies/μL (Figure 6B). Compared with the strip assay, the fluorescence assay exhibited higher sensitivity. When DNA from APP, Pm, GPS, Salmonella, EPEC, S. aureus, A. hydrophila was used as templates, both the optimized RPA-PfAgo-RTF and RPA-PfAgo-LFD assays exclusively detected *S. suis*, with no cross-reactivity to other pathogens (Figures 6C, D).

### 3.7 Clinical sample tests of the RPA-PfAgo-RTF and RPA-PfAgo-LFD assays

**Figure 7:**
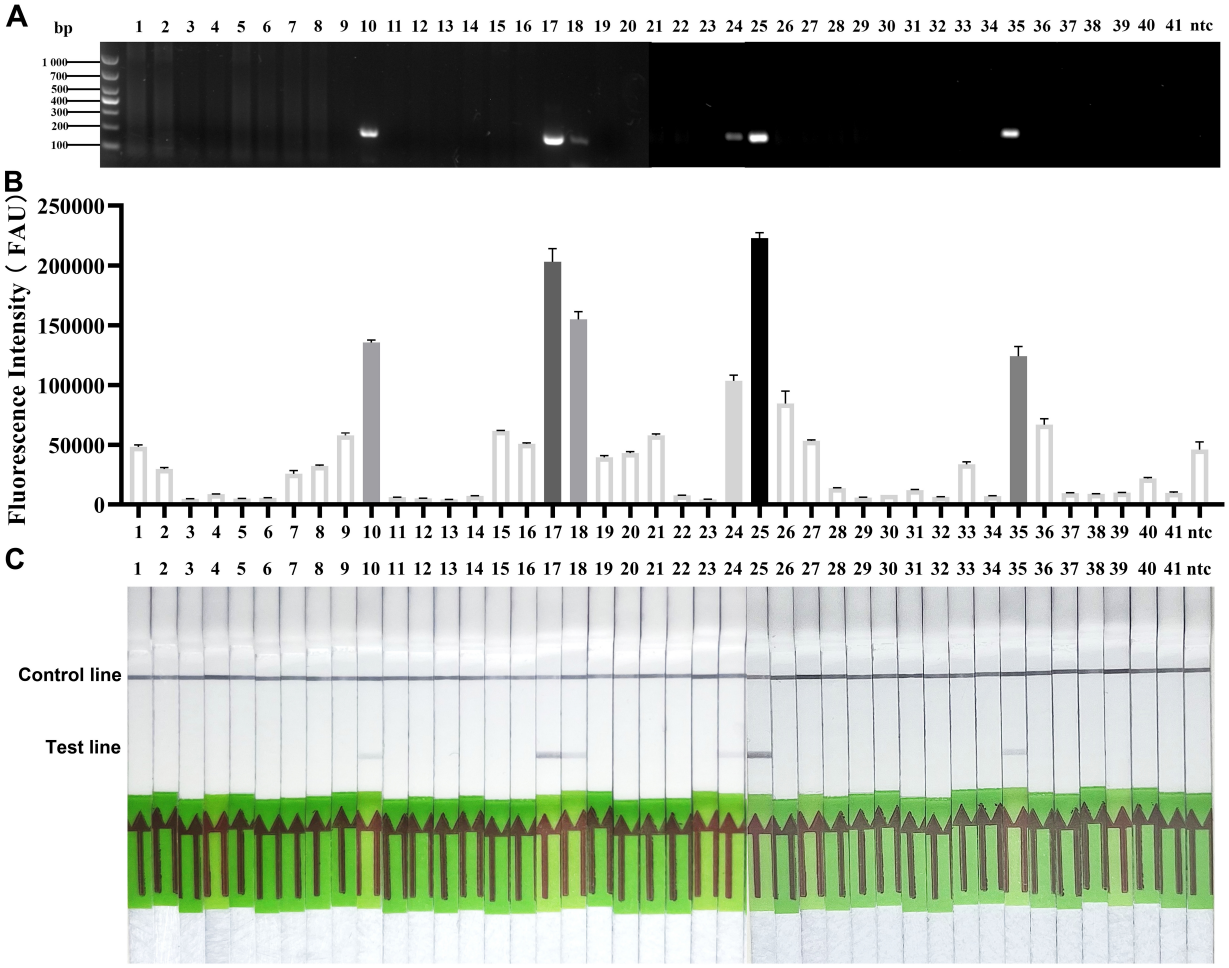
Detection results of clinical samples for SS2. **(A)** The results of the PCR analysis of samples collected. **(B)** The results of the RPA-PfAgo-RTF detection of samples collected. **(C)** The results of the RPA-PfAgo-LFD detection of samples collected. 1–41: Test samples, ntc: no-template control.

A total of 41 clinical samples were tested and analyzed. As shown in Figure 7, both the RPA-PfAgo fluorescence assay and the RPA-PfAgo-LFD assay detected six SS2-positive samples (two blood samples, one lung tissue sample, and three tonsil tissue samples) and 35 negative samples (18 blood samples, 10 lung tissue samples, and seven tonsil tissue samples). These results were fully consistent with those obtained using the standard PCR assay, thereby underscoring the reliability, robustness, and clinical applicability of the RPA-PfAgo fluorescence and RPA-PfAgo-LFD methods established in this study.

## 4 Discussion

Streptococcus suis is a significant bacterial pathogen affecting swine. Among its various serotypes, serotype 2 (SS2) is considered the most virulent and is capable of causing a wide range of infections in both humans and animals (1, 2). SS2 infection causes severe diseases in pigs, such as meningitis, arthritis, and sepsis. Its pathogenicity is also closely linked to increasing antibiotic resistance (3, 4). Epidemiological data indicate that SS2 constitutes a major proportion of Streptococcus suis infections in many countries and regions. Notably, in China, the isolation rate of SS2 from pig herds reaches 16.9% (25). With the growing challenge of antibiotic resistance, the clinical management of SS2 has become increasingly complex, resulting in a rise in the severity of infection cases (13, 14). Therefore, continuous surveillance and epidemiological investigation of SS2 are essential to monitor its transmission trends and assess its impact on public health. In response to this need, this study developed two rapid, sensitive, and specific detection methods for SS2: RPA-PfAgo-RTF and RPA-PfAgo-LFD. Both methods can deliver results within one hour. When evaluated using 41 clinical samples, the results obtained from these two systems were fully consistent with those of the national standard PCR test, confirming their reliability in diagnosing SS2 infections.

The *cps2J* gene, a key component of the capsular polysaccharide (CPS) synthesis gene cluster in *Streptococcus suis* serotype 2, exhibits a high degree of sequence conservation among strains (26, 27). Genomic analyses have revealed that *cps2J* is present as a single-copy gene in the *S. suis* genome, a feature that provides a robust basis for its application in molecular diagnostics (28). The single-copy nature of this gene ensures that its copy number remains unaffected by variations in culture conditions, thereby enhancing the reliability of quantitative detection results (29). All three assays targeting the *cps2J* gene showed high specificity, with no cross-reactivity to other *S. suis* serotypes or common porcine pathogens. These results are in agreement with previous studies, suggesting that the *cps2J* gene serves as a reliable molecular marker for the identification of SS2 (30, 31).

Sensitivity analysis revealed that the RPA-PfAgo-RTF method could detect as few as 10^0^ copies/μL, outperforming the RPA-PfAgo-LFD method (10^2^ copies/μL) and conventional PCR in this study. The higher sensitivity is mainly attributed to the PfAgo-mediated secondary cleavage, which greatly enhances the signal-to-noise ratio. This mechanism is consistent with observations reported in other PfAgo-based detection systems (32, 33). Compared with CRISPR-based detection, the PfAgo system does not require PAM recognition and allows single-base discrimination. LAMP offers fully isothermal amplification but requires multiple primers and frequently produces non-specific products. In contrast, RPA-PfAgo provides a higher signal-to-noise ratio and simpler probe design, while maintaining comparable turnaround time (≤1 h) and minimal equipment requirements. Although PfAgo requires a higher operating temperature, only the cleavage step needs to reach 95 °C. This heating requirement can be met easily using portable dry-bath devices commonly used in field diagnostics, and therefore does not hinder the feasibility of deploying the assay in point-of-care settings. Additionally, PfAgo activity was not affected by the Mg^2+^ ions remaining from the upstream RPA reaction. Consistent with previous reports, PfAgo requires Mn^2+^ as its catalytic cofactor, and Mg^2+^ does not competitively inhibit its nuclease activity when Mn^2+^ is present in excess (17, 20). This supports the compatibility of RPA and PfAgo in a single workflow. Although the sensitivity of the LFD-based system is slightly lower than that of conventional methods, it provides notable advantages for field applications due to its independence from specialized equipment and its ability to yield results that can be easily interpreted. This format aligns with the point-of-care testing strategy, which has been effectively implemented in the veterinary diagnosis of various other pathogens (34, 35).

## 5 Conclusions

In conclusion, the RPA-PfAgo-RTF and RPA-PfAgo-LFD assays developed in this study provide fast, highly specific, and operationally simple tools for the detection of Streptococcus suis serotype 2. By integrating isothermal amplification with PfAgo-mediated secondary cleavage, these platforms achieve sensitive identification of SS2 without the need for sophisticated instrumentation, making them suitable for both laboratory diagnostics and on-farm point-of-care testing.

Importantly, the dual-format design (fluorescence and LFD) enhances adaptability across diverse testing environments, supporting rapid decision-making during herd surveillance, outbreak investigation, and routine health monitoring. While further validation using large-scale field samples and multiplexing strategies is warranted, the current findings demonstrate strong potential for incorporating RPA-PfAgo assays into veterinary diagnostic workflows and advancing rapid pathogen detection in swine health management.

## Data Availability

The raw data supporting the conclusions of this article will be made available by the authors, without undue reservation.

## References

[1] Feng Y Zhang H Wu Z Wang S Cao M Hu D . Streptococcus suis infection: an emerging/reemerging challenge of bacterial infectious diseases? Virulence. (2014) 5:477–97. doi: 10.4161/viru.28595 24667807 PMC4063810

[2] Gottschalk M Segura M. The pathogenesis of the meningitis caused by streptococcus suis: the unresolved questions. Vet Microbiol. (2000) 76:259–72. doi: 10.1016/S0378-1135(00)00250-9 10973700

[3] Goyette-Desjardins G Auger JP Xu J Segura M Gottschalk M. Streptococcus suis, an important pig pathogen and emerging zoonotic agent-an update on the worldwide distribution based on serotyping and sequence typing. Emerg Microbes Infect. (2014) 3:e45. doi: 10.1038/emi.2014.45 26038745 PMC4078792

[4] Lun ZR Wang QP Chen XG Li AX Zhu XQ. Streptococcus suis: an emerging zoonotic pathogen. Lancet Infect Dis. (2007) 7:201–9. doi: 10.1016/S1473-3099(07)70001-4 17317601

[5] Niazy M Hill S Nadeem K Ricker N Farzan A. Compositional analysis of the tonsil microbiota in relationship to streptococcus suis disease in nursery pigs in ontario. Anim Microbiome. (2022) 4:10. doi: 10.1186/s42523-022-00162-3 35063043 PMC8780311

[6] Lu Y Li S Shen X Zhao Y Zhou D Hu D . The type ii histidine triad protein htpsc facilitates invasion of epithelial cells by highly virulent streptococcus suis serotype 2. J Microbiol. (2021) 59:949-957. doi: 10.1007/s12275-021-1129-1 34491523

[7] Zhang S Duan M Li S Hou J Qin T Teng Z . Current status of recombinase polymerase amplification technologies for the detection of pathogenic microorganisms. Diagn Microbiol Infect Dis. (2023) 108:116097. doi: 10.1016/j.diagmicrobio.2023.116097 39491865

[8] Wang H Dong C Tian X Pan Y Wang L An T . Development and application of a dual lamp-lfd assay for the simultaneous detection of streptococcus suis and glaesserella parasuis. Front Cell Infect Microbiol. (2025) 15:1575365. doi: 10.3389/fcimb.2025.1575365 40235932 PMC11996922

[9] Lai R Yang C Wu L Wu W Li L Liu Wet al. Development and implementation of a taqman triplex real-time pcr assay for concurrent detection of pseudorabies virus, porcine teschovirus 1, and streptococcus suis 2. Front Vet Sci. (2025) 12:1589175. doi: 10.3389/fvets.2025.1589175 40607354 PMC12213347

[10] Yang J Li W Hu Y Han Y Lei C Wang H. Establishment of a rapid raa-crispr/cas12a system targeting the recn gene for on-site detection of streptococcus suis in livestock and fresh pork meat. Funct Integr Genomics. (2025) 25:99. doi: 10.1007/s10142-025-01605-1 40327171

[11] Okwumabua O O'Connor M Shull E. A polymerase chain reaction (pcr) assay specific for streptococcus suis based on the gene encoding the glutamate dehydrogenase. Fems Microbiol Lett. (2003) 218:79-84. doi: 10.1111/j.1574-6968.2003.tb11501.x 12583901

[12] Marois C Le Devendec L Gottschalk M Kobisch M. Detection and molecular typing of streptococcus suis in tonsils from live pigs in france. Can J Vet Res. (2007) 71:14–22. 17193877 PMC1635993

[13] Li Y Ma B Jia X Wan Y Ni S Chen G . Population genomics, virulence traits, and antimicrobial resistance of streptococcus suis isolated in china. Microorganisms. (2025) 13:1197. doi: 10.3390/microorganisms13061197 40572085 PMC12195111

[14] Xia Y Wang Z Hu Y Zhao P Li J Zhang L . Isolation, identification, genomic diversity, and antimicrobial resistance analysis of streptococcus suis in hubei province of china from 2021 to 2023. Microorganisms. (2024) 12:917. doi: 10.3390/microorganisms12050917 38792744 PMC11124115

[15] Chen W Zhang J Wei H Su J Lin J Liang X . Rapid and sensitive detection of methicillin-resistant staphylococcus aureus through the rpa-pfago system. Front Microbiol. (2024) 15:1422574. doi: 10.3389/fmicb.2024.1422574 39234537 PMC11371615

[16] Piepenburg O Williams CH Stemple DL Armes NA. Dna detection using recombination proteins. Plos Biol. (2006) 4:e204. doi: 10.1371/journal.pbio.0040204 16756388 PMC1475771

[17] Swarts DC Jore MM Westra ER Zhu Y Janssen JH Snijders AP . Dna-guided dna interference by a prokaryotic argonaute. Nature. (2014) 507:258–61. doi: 10.1038/nature12971 24531762 PMC4697943

[18] Liao JY Feng XY Zhang JX Yang TD Zhan MX Zeng YM . Rt-rpa-pfago detection platform for one-tube simultaneous typing diagnosis of human respiratory syncytial virus. Front Cell Infect Microbiol. (2024) 14:1419949. doi: 10.3389/fcimb.2024.1419949 39119294 PMC11306018

[19] Chen L Chen W Wei H Lin W Zhang C Hu H . Rapid and supersensitive allele detection of plasmodium falciparum chloroquine resistance via a pyrococcus furiosus argonaute-triggered dual-signal biosensing platform. Parasit Vectors. (2024) 17:488. doi: 10.1186/s13071-024-06575-0 39582041 PMC11587582

[20] Sheng G Zhao H Wang J Rao Y Tian W Swarts DC . Structure-based cleavage mechanism of thermus thermophilus argonaute dna guide strand-mediated dna target cleavage. Proc Natl Acad Sci U S A. (2014) 111:652–57. doi: 10.1073/pnas.1321032111 24374628 PMC3896195

[21] Zhao Y Yang M Zhou C Guo B Wang K Song C . Establishment of a simple, sensitive, and specific asfv detection method based on pyrococcus furiosus argonaute. Biosens Bioelectron. (2024) 254:116230. doi: 10.1016/j.bios.2024.116230 38520983

[22] Zhao Y Zhang T Zhou C Guo B Wang H . Pyrococcus furiosus argonaute based detection assays for porcine deltacoronavirus. Acs Synth Biol. (2024) 13:1323–31. doi: 10.1021/acssynbio.4c00045 38567812

[23] Iorio R Biadasz N Giunta N Chen AF Einhorn TA Karia R. A digital platform for the self-management of knee arthritis: myarthritisrx.com. Orthop Clin North Am. (2023) 54:1–6. doi: 10.1016/j.ocl.2022.08.005 36402505

[24] Lin L Luo Q Li L Zheng Y Wei H Liao J . Recombinase polymerase amplification combined with pyrococcus furiosus argonaute for fast salmonella spp. Testing in food safety. Int J Food Microbiol. (2024) 417:110697. doi: 10.1016/j.ijfoodmicro.2024.110697 38642433

[25] Zhang B Ku X Yu X Sun Q Wu H Chen F . Prevalence and antimicrobial susceptibilities of bacterial pathogens in Chinese pig farms from 2013 to 2017. Sci Rep. (2019) 9:9908. doi: 10.1038/s41598-019-45482-8 31289289 PMC6616368

[26] Okura M Osaki M Nomoto R Arai S Osawa R Sekizaki T . Current taxonomical situation of streptococcus suis. Pathogens. (2016) 5:45. doi: 10.3390/pathogens5030045 27348006 PMC5039425

[27] Smith HE Vecht U Wisselink HJ https://pubmed.ncbi.nlm.nih.gov/?term=%22Stockhofe-Zurwieden%20N%22%5BAuthor%5DStockhofe-Zurwieden N, Biermann Y, https://pubmed.ncbi.nlm.nih.gov/?term=%22Smits%20MA%22%5BAuthor%5DSmits MAet al. Mutants of streptococcus suis types 1 and 2 impaired in expression of muramidase-released protein and extracellular protein induce disease in newborn germfree pigs. Infect Immun. (1996) 64:4409–12. doi: 10.1128/iai.64.10.4409-4412.1996 8926123 PMC174391

[28] Athey TB Auger JP Teatero S Dumesnil A Takamatsu D Wasserscheid J . Complex population structure and virulence differences among serotype 2 streptococcus suis strains belonging to sequence type 28. Plos One. (2015) 10:e137760. doi: 10.1371/journal.pone.0137760 26375680 PMC4574206

[29] Feng Y Zhang H Ma Y Gao GF. Uncovering newly emerging variants of streptococcus suis, an important zoonotic agent. Trends Microbiol. (2010) 18:124–31. doi: 10.1016/j.tim.2009.12.003 20071175

[30] Eiamphungporn W Laohabutr P Kaewsai N Pornsuwan S Yainoy S Chatupheeraphat C . Development of a recombinase polymerase amplification nucleic acid lateral flow assay for detecting streptococcus suis serotype 2 in pork. Sci Rep. (2025) 15:10442. doi: 10.1038/s41598-025-95480-2 40140541 PMC11947096

[31] Nga TV Nghia HD Tu LT Nga TV Nghia HD Tu le TP . Real-time pcr for detection of streptococcus suis serotype 2 in cerebrospinal fluid of human patients with meningitis. Diagn Microbiol Infect Dis. (2011) 70:461–7. doi: 10.1016/j.diagmicrobio.2010.12.015 21767702 PMC3146703

[32] Zhao Y Su D Li M Deng Q Xiong S Feng Y . Application of pyrococcus furiosus argonaute coupled with rpa for highly sensitive and specific detection of mycoplasma gallisepticum. Poult Sci. (2025) 104:105116. doi: 10.1016/j.psj.2025.105116 40188619 PMC12001110

[33] Zhao C Yang L Zhang X Tang Y Wang Y Shao X . Rapid and sensitive genotyping of SARS-cov-2 key mutation l452r with an rpa-pfago method. Anal Chem. (2022) 94:17151–59. doi: 10.1021/acs.analchem.2c03563 36459151

[34] Mao X Ma Y Zhang A Zhang L Zeng L Liu G. Disposable nucleic acid biosensors based on gold nanoparticle probes and lateral flow strip. Anal Chem. (2009) 81:1660–68. doi: 10.1021/ac8024653 19159221

[35] Posthuma-Trumpie GA Korf J van Amerongen A. Lateral flow (immuno)assay: its strengths, weaknesses, opportunities and threats. A literature survey. Anal Bioanal Chem. (2009) 393:569–82. doi: 10.1007/s00216-008-2287-2 18696055

